# Resistance Mechanisms of Plant Pathogenic Fungi to Fungicide, Environmental Impacts of Fungicides, and Sustainable Solutions

**DOI:** 10.3390/plants13192737

**Published:** 2024-09-30

**Authors:** Tarequl Islam, Danishuddin, Noshin Tabassum Tamanna, Muhammad Nurul Matin, Hasi Rani Barai, Md Azizul Haque

**Affiliations:** 1 Department of Microbiology, Noakhali Science and Technology University, Noakhali 3814, Bangladesh; tarequembg@gmail.com; 2 Department of Biotechnology, Yeungnam University, Gyeongsan 38541, Republic of Korea; danish23@yu.ac.kr (D.); nmatin2@yahoo.com (M.N.M.); 3 Department of Pharmacy, Noakhali Science and Technology University, Noakhali 3814, Bangladesh; tamannanoshin@gmail.com; 4 Professor Joarder DNA and Chromosome Research Laboratory, Department of Genetic Engineering and Biotechnology, University of Rajshahi, Rajshahi 6205, Bangladesh; 5 School of Mechanical and IT Engineering, Yeungnam University, Gyeongsan 38541, Republic of Korea

**Keywords:** emerging phytopathogens fungi, antimicrobial resistance, environmental changes, biocontrol agents, antimicrobial peptides

## Abstract

The significant reduction in agricultural output and the decline in product quality are two of the most glaring negative impacts caused by plant pathogenic fungi (PPF). Furthermore, contaminated food or transit might introduce mycotoxins produced by PPF directly into the food chain. Eating food tainted with mycotoxin is extremely dangerous for both human and animal health. Using fungicides is the first choice to control PPF or their toxins in food. Fungicide resistance and its effects on the environment and public health are becoming more and more of a concern, despite the fact that chemical fungicides are used to limit PPF toxicity and control growth in crops. Fungicides induce target site alteration and efflux pump activation, and mutations in PPF result in resistance. As a result, global trends are shifting away from chemically manufactured pesticides and toward managing fungal plant diseases using various biocontrol techniques, tactics, and approaches. However, surveillance programs to monitor fungicide resistance and their environmental impact are much fewer compared to bacterial antibiotic resistance surveillance programs. In this review, we discuss the PPF that contributes to disease development in plants, the fungicides used against them, factors causing the spread of PPF and the emergence of new strains, the antifungal resistance mechanisms of PPF, health, the environmental impacts of fungicides, and the use of biocontrol agents (BCAs), antimicrobial peptides (AMPs), and nanotechnologies to control PPF as a safe and eco-friendly alternative to fungicides.

## 1. Introduction

Globally, phytopathogens represent a major threat to crop productivity. Plant infections caused by fungi, bacteria, viruses, and nematodes seriously impair or destroy crops, significantly lowering the quantity and quality of agricultural products. Each year, these losses pose a danger to the world’s food supply [1,2]. Furthermore, pathogenic infections in the field or during post-harvest storing can be harmful for livestock and people, especially if the disease produces toxins in or on edible goods [2].

In recent decades, fungi have been responsible for an increasing number of epidemics affecting humans, plants, and animals [3]. This issue is intensified by the growing resistance of pathogenic organisms to antifungal medications. When fungi are exposed to fungicides in the environment over time, resistance develops [4]. Fungal diseases such as *Aspergillus fumigatus* are evolving novel resistant forms, while newly emerging fungal species like *Candida auris* and *Trichophyton indotineae* are exhibiting antifungal resistance profiles [3,5].

New disease outbreaks can result from alterations in fungal survival, infectivity, and host vulnerability caused by changes in the climate [6]. Additionally, these changes might encourage the establishment of novel virulent strains, which is extremely concerning for future epidemiological studies [7]. Numerous studies on the relationship between fungal infections and climate change have demonstrated that fungal diseases have become more prevalent as a result of climate change, and they are now widely acknowledged as a global threat to significant crops [6,8,9,10]. Fungi require adaptation or community shifts to survive in the face of land-use change, drought, and climate fluctuation; however, certain fungi with higher thermal optima are more resilient to these changes [11].

Plant pathogens are spreading outside their typical geographical areas due to globalization and worldwide trade, with the potential to adapt to climatic and environmental changes and contemporary agricultural practices, like altered land uses and the widespread use of antifungal agents [12]. Despite their potential to cause severe damage to economically significant crops, emerging fungal diseases are still poorly understood and understudied, posing a growing threat to bionetworks, overall health, food safety, and the economy [13]. Although many questions remain about how these fungal pathogens spread, evolve, adopt novel ecological strategies, switch hosts, and cause infections, these emerging pathogens have the potential to serve as “true reservoirs” for future disease epidemics [10]. Published data suggest that common saprophytic fungi from the *Aspergillus*, *Penicillium*, and *Cryptococcus* genera are emerging as possible plant pathogens. During cultural practices or the post-harvest/storage stages, these fungi can pose a significant hazard to staple crops, including rice, wheat, maize, and potatoes [14]. The potential impact on global food security could be significant if specific disease management policies are not implemented and these infections are not promptly and accurately identified [13].

Controlling various plant pathogens necessitates an effective management system to ensure sufficient food supply for the world’s growing population [15,16]. At present, the main approaches for controlling plant illnesses include the development of disease-resistant crops, the use of chemical pesticides, and the implementation of effective practices. Over the last few decades, these methods have been fundamental in growing yield and quality [17]. Unfortunately, the abuse of chemical pesticides has led to environmental degradation, which has limited their use in agriculture [17,18]. As an eco-friendly and sustainable way of controlling crop diseases, researchers are now considering the use of beneficial microorganisms (e.g., bacterial and fungal endophytes) [17,19]. Different bacteria have shown great potential as BCAs for various plant pathogens. It has also been proven that fungi play a crucial role in preventing serious diseases in essential crops [19,20]. Significant emphasis has also been placed on research into fungal strains such as BCAs for plant disease control. These fungus-based BCAs have the potential to be used as biopesticides in field or greenhouse studies, as they exhibit strong antagonistic activity against various types of soil- and airborne plant pathogens [19,21].

In this review, we briefly discuss the role of PPF in disease development in plants, factors contributing to the spread of PPF in the environment and plants, and the emergence of new pathogenic fungal strains. We also cover the antibiotic resistance mechanisms of PPF, the health and environmental impacts of chemical fungicides, and the use of BCAs to combat PPF as a safe and eco-friendly alternative to antibiotics. Furthermore, we examine the use of AMPs from various natural sources as potential antifungal agents and recent advances in nanotechnology to combat PPF. Nanoparticles (NPs) offer many advantages over chemical antibiotics, such as efficiently reaching target sites and being less prone to developing resistance.

## 2. Control of PPF by Antibiotics

### 2.1. Fungal Pathogens and Diseases

There are a lot of PPF infecting plants and crops. Listing or ranking all of them is a troublesome task. However, the journal *Molecular Plant Pathology* ranked the top 10 fungal pathogens based on a survey conducted with the participation of 495 plant pathologists worldwide [22]. These fungal pathogens include (1) *Magnaporthe oryzae*, (2) *Botrytis cinerea*, (3) *Puccinia* spp., (4) *Fusarium graminearum*, (5) *F. oxysporum*, (6) *Blumeria graminis*, (7) *Mycosphaerella graminicola*, (8) *Colletotrichum* spp., (9) *Ustilago maydis*, and (10) *Melampsora lini*. Other important PPF that are not included in the above list are *Phakopsora pachyrhizi* and *Rhizoctonia solani* [22].

New pathogens brought about by environmental changes and assessment are known as emerging pathogens [10]. One such emerging pathogen is *Neopseudocercosporella capsellae*, which causes white leaf spots on Brassicaceae plants. Recent reports indicate that this fungus is harming commercially significant Brassicaceae crops, such as oilseed rape, vegetables, condiments, and feed brassicas [23]. Before infection occurs, *N. capsellae* can transition between two morphologies (septate hyphae and single-celled yeast phase) on various artificial culture mediums (in vitro) or in planta on the host surface. It has been demonstrated that the hyphae-to-yeast transformation involves the development of arthrospores (arthroconidia) and two morphologically distinct types of blastospores (blastoconidia): meso- and micro-blastospores [24].

Numerous crop diseases are caused by *Bipolaris sorokiniana* [8]. In China, hazelnuts (*Corylus heterophylla*), a significant nut crop, are reportedly declining due to fungal branch canker and dieback. Three *Cytospora* and two *Diaporthe* species were identified as the causative agents through morphological observations combined with multi-locus phylogenetic analysis [25]. Additionally, this epidemic has revealed three new species: *C. corylina*, *C. curvispora*, and *D. corylicola*. Two recognized species, *C. leucostoma* and *D. eres*, are also documented in this study [25].

### 2.2. Factors in Spreading PPF

Food security and agricultural practices are significantly impacted by climate change, which could have detrimental effects on several economically significant crops sooner or later. Perennial crops, like tea, are especially susceptible. Crop-associated fungal infections will likely also be impacted by climate change [9]. For the vector hosts, an intricate combination of several environmental factors (e.g., temperature and moisture) produces the ideal habitat for the pathogens. Global warming and climate change will have catastrophic repercussions on environments that sustain animal, plant, and human life. Pathogens, especially those of overlooked tropical illnesses, are predicted to resurface and develop in several countries, including North America and Europe [9,12]. By 2050, baseline tea-growing areas will no longer be suitable for *Camellia sinensis* var. *assamica* (32–34% loss) and *C. sinensis* var. *sinensis* (15–32% loss), according to models of the climate-fungal pathogen [9]. In the regions where tea is currently being grown, issues will arise from both known and possibly unknown fungal diseases [9].

Grasslands constitute a significant ecological and economic element of the planet’s vegetation, and the stability of these ecosystems is greatly influenced by the fungal populations that inhabit them. Grass–fungi interactions can be mutualistic or harmful. It is well recognized that fungi, insects, various grassland animals, and herbivorous mammals all contribute significantly to preserving the variety and biomass of grasslands [26]. Because pathogenic fungi alter the physiology and chemical composition of grasses, they have a considerable impact on the population biology of grasses and their contribution to plant communities [26,27].

In addition to the increasing reliance on fossil fuels and water, intensive agriculture has been linked to biodiversity losses [28]. Crop disease protection in our modern agroecosystems is primarily provided by cultivars having single main R genes and single-target-site fungicides. However, this defense mechanism is proving to be temporary, as monocultures encourage the growth of novel fungal strains that may overcome host resistance and resist commonly used fungicides, providing agricultural pathogens with the ideal environment for reproduction and proliferation [29]. Furthermore, when exposed to antibiotics, microbes are likely to develop resistance against them [30,31,32,33]. The widespread use of chemical fungicides is another factor contributing to the environmental development of novel PPF [28,34].

The continual existence of host tissue, especially in temperate zones, also sustains fungal populations [35]. Thus, *Zymoseptoria tritici* exhibits every trait associated with a high potential for evolution: great variety, frequent sexual reproduction, sizable effective populations, and high rates of recombination and mutation [35]. This promotes the development of novel characteristics that enable *Z. tritici* to flourish on novel host varieties, in novel environmental circumstances, or in the face of extensive fungicide use [36]. The most damaging of these is soybean rust (SBR), which is brought on by the fungus *P. pachyrhizi*. In areas where disease conditions are favorable, losses of up to 80% are not uncommon [37,38]. One reason for the severity and spread of SBR is the prolific production of asexual spores, which leads to recurrent cycles of infection [39].

There is ample evidence of the spread of wheat blast to Asia’s wheat-producing regions, especially Bangladesh. Here, crop losses due to wheat blast have reached 50% [40,41]. A common characteristic of disease introduction in a new geographic location is such significant devastation. The reason behind such severe epidemics is frequently the pathogen’s migration to new areas, where it may not be affected by viruses or competitors. Their climate might be more suited to the areas, enabling prolonged intervals of maximal pathogen virulence or enhanced survival in between host growth seasons. Most crucially, though, newly discovered regions contain naive hosts that are not aware of cues to develop an anti-pathogen response since they have not co-evolved with the pathogen [38]. If the velocity of pathogens’ migration is pushed beyond the frequency at which host defense systems evolve, the likelihood of host naivety is significantly raised. This can occur spontaneously when certain diseases move large distances quickly through the atmosphere, as *P. pachyrhizi* demonstrates [38].

### 2.3. Antifungal Agrochemicals in PPF Control

By 2050, it is predicted that the world’s population will have increased by roughly 30%, necessitating investments to boost productivity and output in agriculture [42]. Consequently, it is essential to employ effective fungicides to safeguard crops against disease during both the large-scale agricultural production phase and the post-harvest phase [43]. The agricultural industry has faced a number of difficulties recently, including lower crop yields brought on by abiotic stressors, diseases, and pests.

Antifungal agrochemicals are chemicals used to kill PPF in crops. These are frequently employed in agricultural systems to prevent illness and maintain crop quality and output. There are different classes of antifungal agrochemicals used to control PPF. All of these chemicals have specific modes of action and specificity. For example, azoles, especially triazoles, are highly efficient against a broad range of plant diseases such as leaf rust, leaf spots, and powdery mildew, inhibiting fungal ergosterol biosynthesis by impeding 14α-demethylase [44,45,46,47]. Another major class of antibiotics is echinocandins (anidulafungin, caspofungin, and micafungin), which inhibit 1,3-Beta-glucan synthase in fungal cells. As a result, the formation of glucan in the fungal cell wall is blocked, resulting in the death of fungal cells [48]. Other classes of antifungal agrochemicals used in agriculture include polyenes, allylamines, morpholine, and anilinopyrimidine [42]. An organomercurial compound, the first organic fungicide, was created at the start of the 20th century. Several fungicides that are effective against fungi like *Fusarium* spp. and *Dreschlera* spp. were commercialized as a result of further research in this area, including 2-methoxyethyl silicate and 2-hydroxyphenyl mercury [49]. Table 1 shows some common fungal diseases of crops and the antibiotics found to be effective against these PPF, while Table 2 shows the mode of action of different fungicides on PPF.

## 3. Mechanisms of Antifungal Resistance

Every group of fungicides has its mode of action, as shown in Table 3. To develop resistance, fungal pathogens develop their own ways to either block the target sites of fungicides, overexpress efflux pumps, or undergo genetic modifications [5]. One typical mechanism leading to acquired resistance to antifungal chemicals is drug target change. Resistance to azoles is caused by changes in the genes that encode 14-α-demethylase, the target of azole fungicides (*cyp51* in molds and *erg11* in yeast). Several PPF, such as *Penicillium digitatum*, *Cercospora beticola*, *Erysiphe necator*, *B. cinérea*, *M. graminicola*, *O. acuformis*, and *Podosphaera fusca*, have been reported to develop resistance against azoles through target modification [5,76,77,78]. Unbelievably, azole-using environments are becoming increasingly contaminated with *cyp51A* gene promoter duplications paired with specific amino acid substitutions TR34/L98H and TR46/Y121F/T289A. These duplications have also been isolated from hospital patients who have never taken antifungal medications before [79,80,81,82]. Through recombination between resistant and susceptible isolates, a single introduction of a strain bearing the TR34/L98H mutation appears to have resulted in extensive dissemination throughout susceptible Aspergillus populations, according to recent genomic research of *A. fumigatus* isolates from the USA [83]. Another common way that azole resistance is acquired is through increased expression of the drug target. There are some transcription regulators that contribute to the overexpression of cyp51A/ERG11, resulting in azole resistance. In *A. fumigatus* and *C. neoformans*, the sterol regulatory element-binding protein (SREBP), SrbA, and Sre1 control the expression of cyp51A/ERG11 when exposed to azole [84,85]. Additionally, the transcription factors AtrR and SltA also regulate the expression of cyp51A in *A. fumigatus*, which is responsible for azole resistance [86,87,88]. Interestingly, AtrR induces the expression of *cdr1B*, an ABC transporter implicated in azole resistance. Therefore, it may be claimed that AtrR, through co-regulating the drug target (Cyp51A) and putative drug efflux pump (Cdr1B), plays a crucial role in a novel azole resistance mechanism [86]. Similarly, mutations in the genes encoding the therapeutic targets *FKS1* (*Candida*, *Cryptococcus*, and *Aspergillus* species) and *FKS2* (*C. glabrata* alone) are often responsible for resistance to echinocandin therapy [89,90]. Although polyene resistance is uncommon, changes in the ergosterol production pathway can lead to ergosterol depletion and the build-up of alternative sterols. Numerous ergosterol biosynthesis genes, including *ERG2*, *ERG3*, *ERG6*, and *ERG11*, have been linked to mutations in *Candida* species that exhibit resistance to amphotericin B [91,92,93].

A common mechanism of resistance to various antifungal medications is accelerated drug efflux, which operates alongside target modification or overexpression. The overexpression of plasma membrane efflux pumps, reducing intracellular drug accumulation, is frequently associated with resistance to azole therapy. Conversely, efflux-mediated resistance mechanisms are rarely implicated in adaptive resistance to echinocandins and polyenes, as they are weak pump substrates [94]. Constitutive overexpression of the ABC-transporter genes *CDR1* and *CDR2* results from gain-of-function mutations in the transcriptional activator gene *TAC1*, while activating mutations in *MRR1* cause the upregulation of *MDR1* [95,96]. Similarly, the clinical resistance of *C. auris* to fluconazole is influenced by gain-of-function mutations in *TAC1B* and *MRR1A* [97,98]. Thus, various fungal infections demonstrate efflux-mediated resistance to azoles, highlighting the potential utility of targeting drug efflux to overcome resistance.

Genomic modifications are another mechanism by which fungal infections develop resistance to fungicides. Azole-resistant clinical isolates and laboratory-derived resistant strains of *Candida albicans* have been observed to duplicate the left arm of chromosome 5, forming an isochromosome (i5(L)) [99]. This duplication leads to the overexpression of azole target protein Erg11 and the transcription factor Tac1, which controls drug efflux [100]. Recent studies in *C. albicans* have revealed that exposure to the endoplasmic reticulum stress-inducing drug tunicamycin or the cancer treatment hydroxyurea selects for trisomy of chromosome 2, conferring tolerance to the echinocandin caspofungin [101,102]. While at least three genes on chromosome 2 (*ALG7*, *RTA2*, and *RTA3*) affect only tunicamycin responses and not caspofungin, extra copies of *MKK2* affect tolerance to both fungicides. Other chromosome 2 genes (*RNR1* and *RNR21*) do not impact tunicamycin or caspofungin tolerance, but they do affect hydroxyurea tolerance [101]. Aneuploidy can confer resistance to medications unrelated to cross-tolerance by altering the copy number of numerous genes simultaneously [102]. Genomic rearrangements such as chromosome translocations, segmental duplications, and the sporadic creation of de novo mini-chromosomes underlie azole resistance in the haploid yeast *C. glabrata* [103]. Heteroresistance in *C. neoformans* is frequently acquired through disomy formation, with chromosome 1, containing both the ABC transporter genes *AFR1* and *ERG11*, being the most common disomic chromosome [104]. Additionally, recent findings suggest that *A. fumigatus* develops triazole resistance through horizontal gene transfer (HGT) [105]. Under voriconazole stress alone, *A. fumigatus* can develop resistance via both chromosomal and plasmid-mediated HGT, indicating that HGT may contribute to antifungal resistance in this pathogen [105]. Table 3 summarizes the antifungal resistance mechanisms of various PPF against different classes of fungicides.

**Table 3:** Fungicide resistance mechanisms of PPF.

Fungicide Classes	Example	Resistant Fungal Pathogens	Mechanisms of Resistance	References
Polyenes	Amphotericin B, Candicidin, Hamycin, Natamycin, Nystatin, Rimocidin	*Aspergillus* spp., *C. albicans*, *C. glabrata*, *C. auris*, *C. neoformans*, *Fusarium* spp.	Target alteration by AmB resistance, *ERG* gene mutation Mutations in *FCY1*, *FCY2*, and *FUR1* genes	[5,76]
Benzimidazoles	Carbendazim, Thiabendazole	*B. cinerea*	Point mutations in the β-tubulin gene resulting in altered benzimidazole-binding site	[77,78]
	Benomyl	*Venturia inaequalis*, *V. pirina, Monilinia fructicola*, *Sclerotinia homoeocarpa*, *Penicillium*	Point mutations in the β-tubulin gene (mutation at position 198)	[106]
SDHIs	Bixafen, Boscalid, Carboxin, Fluaxapyroxad, Fluopyram, Isopyrazam, Penthiopyrad, Sedaxane	*B. cinerea*, *Corynespora cassicola*, *D. bryoniae*, *Botrytis elliptica*, *Podosphaera xanthii*, *P. pachyrhizi*, *Stemphylium botryosum*, *Aspergillus flavus*, *Alternaria alternata*	Mutations in *sdh* genes	[73,107,108,109,110,111,112]
QoIs	Pyraclostrobin, Azoxystrobin, Mandestrobin, Kresoxim-Methyl, Dimoxystrobin, Famoxadone, Fluoxastrobin, Fenamidone, Pyribencarb	*B. cinerea*, *P. pachyrhizi*	Mutation in *cyt*b	[108,111]
Morpholines	Aldomorph, Enpropimorph, Dodemorph, Tridemorph, Fenpropimorph, Fenpropidin Spiroxamine,	*E. necator*, *E. graminis*, *Sphaerotheca fuliginea,*	Unknown	[113,114]
Anilinopyrimidines	Cyprodinil, Mepanipyrim, Pyrimethanil	*B. cinerea*, *V. inaequalis,*	All identified resistance-conferring genes encode proteins that are involved in mitochondrial processes, suggesting that anilinopyrimidines primarily target the mitochondria.	[115,116,117]
DMIs	Cyproconazole, Propiconazole, Tebuconazole	*P. pachyrhizi*	Mutation on *cyp51* gene	[111]
Azoles	Abafungin, Bifonazole, Butoconazole, Lotrimazole, Econazole, Fenticonazole, Ketoconazole, Miconazole, Oxiconazole, Sulconazole, Albaconazole, Inaconazole, Epoxiconazole, Luconazole, Prothioconazole	*Z. tritici*, *V. inaequalis*, *P. digitatum*, *C. beticola*, *M. fructicola*, *E. necator*, *Brumeriela jaapii*, *B. cinérea, B. graminis*, *M. graminicola*, *O. acuformis*, *O. yallundae, P. fusca*	Mutations in *cyp51*, upregulation of *cyp51* and the genes encoding membrane transporters, efflux pump activity, ABC transporter *PMR1* over-expression, mutations in *erg11* or *erg3*	[5,118,119,120]
Echinocandins	Anidulafungin, Aspofungin, Micafungin	*C. albicans*, *C. krusei*, *C. auris*, *C. tropicalis*, *A. fumigatus*	Mutations in *fks1* gene	[4,5]
Others	Azoxystrobin, Difenoconazole	*Alternaria solani*	Mutations in *cyt*b are associated with azoxystrobin resistance	[121]
	Thiophanate-Methyl	*Lasiodiplodia theobromae*	Mutations in β-tubulin gene	[122]

SDHI, succinate dehydrogenase inhibitor; QoI, quinol oxidation inhibitor; DMI, demethylation inhibitor.

## 4. Impact of Antifungal Chemicals on Humans, Plants, and Environment

Fungicide use has significantly increased in major agricultural countries, including China and the USA, by up to 400%. Most fungicides have a degradation half-life ranging from 45 to 120 days, allowing opportunistic fungal pathogens enough time to develop antifungal resistance [123,124,125].

We frequently live in close quarters with opportunistic pathogenic fungi, many of which are capable of producing large amounts of airborne spores. Consequently, a variety of environmental fungal diseases, known as bioaerosols, regularly expose humans to them. While the majority of ambient fungi do not cause discernible pathophysiological events in healthy individuals, those with weakened immune systems or compromised health are more susceptible to a range of disorders, including invasive fungal diseases (IFDs) that can be fatal, allergic, chronic, or superficial [125]. Patients at risk due to immune-suppression drugs (ISDs) include an increasing number of older adults, those whose immune systems have been weakened by COVID-19 or HIV, those undergoing cancer chemotherapy, those with immune suppression therapy required for transplant recipients, or those with influenza [126,127,128].

Numerous fungal infections are acquired from our surrounding habitats, as demonstrated by numerous molecular epidemiological investigations. This is particularly true for IFDs caused by *Coccidioides* spp., *A. fumigatus*, and *Cryptococcus* spp. [81,129,130,131]. Resistance genotypes may also be passed on by humans suffering from invasive fungal diseases (IFDs), while it is unclear how much human and animal activity contributes to environmental antifungal resistance. The incidence of antifungal resistance is predicted to be influenced by several external variables, such as shifting fungicide usage patterns in waste management and the environment [124].

Furthermore, shifting biotic interactions that may involve xenobiotic chemical analogs of fungicides; shifting endangered human host groups, including infections like COVID-19 or influenza; shifting weathers that may change the geographic area of fungi and adaptive landscape for resistance, along with unique means of infection; and shifting virulence of the fungi themselves due to intrinsic genetic changes or synergies with combinations of the above drivers pose risks regarding the spread of pathogens [132,133].

Another significant risk when utilizing antifungal medications is cross-resistance. A previous study showed that fluconazole was ineffective in treating mice infected by previously benomyl-exposed *Cryptococcus gattii* cells because benomyl induced the overexpression of efflux pumps in *C. gattii* [133]. Exposure to fungicides used in agroecosystems has been linked to the establishment of human pathogen isolates resistant to fungicides [43].

There are many helpful microorganisms residing in plants and soil that directly or indirectly support plant growth and provide protection against various infections [2]. However, the use of fungicides on different crops may jeopardize the presence of these microorganisms, especially arbuscular mycorrhizal fungi (AMF) [51]. In a study measuring the effects of azoxystrobin (a systemic broad-spectrum fungicide), flutolanil (a systemic fungicide specific to Basidiomycota), and pencycuron (a contact fungicide specific to *Rhizoctonia*), along with their corresponding formulations (Amistar, Monarch, and Monceren), on the growth and development of the AMF *Rhizophagus irregularis* MUCL 41833 (spore germination, root colonization, extraradical mycelium development, and spore production), it was found that at doses used to control *R. solani*, azoxystrobin and its formulation Amistar inhibited the growth of extraradical mycelium and spore formation at ten times the threshold value [51]. Flutolanil and its derivative Monarch at the suggested dose decreased root colonization and arbuscule development [51].

**Figure 1:**
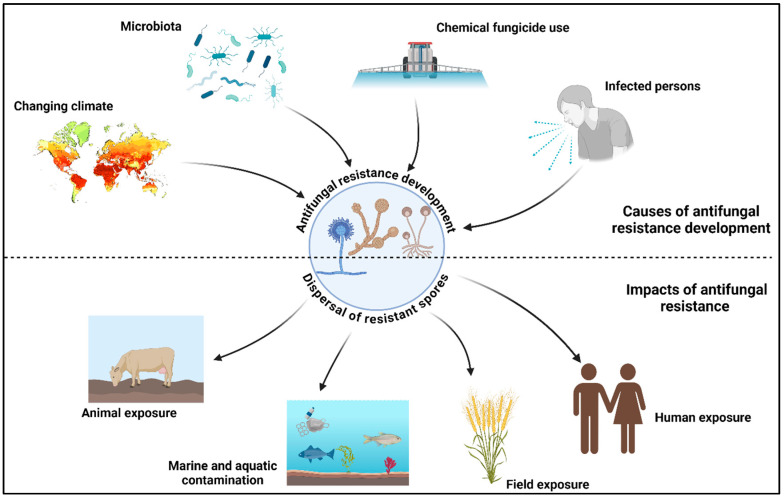
Antifungal resistance development and dispersal of resistant isolates. This figure was created using BioRender software v. 04.

Antifungal medications enter the environment through various pathways (Figure 1). After excretion in urine or feces, active medicinal components from human preparations may release their metabolites into the environment. Environmental contamination also occurs due to pharmaceutical businesses’ wastewater and the improper disposal of unused or expired medications [134]. Fungicides used as plant protection agents have the potential to contaminate soil, groundwater, and surfaces through runoff from agricultural fields and fruit and vegetable processing facilities. They have become serious contaminants in several environmental compartments, including soil, fruits and vegetables, drinking water, municipal sewage, groundwater, and sewage effluent sludge. These drugs and their byproducts may pose potential hazards to humans and other living things in the environment [134]. Additionally, antifungal metals (such as copper) sprayed on plants may lead to the evolution of aquatic microorganisms and the emergence of metal-tolerant microbes [135].

Antifungal medications have been associated with various negative impacts on marine life, including endocrine disruption, reduced larval growth and development, and reproductive abnormalities [136,137,138]. Azole fungicides, in particular, have been linked to disruptions in hormonal balance and effects on the reproductive and physiological systems of aquatic organisms [136].

## 5. BCAs and Nanotechnologies in Controlling PPF

### 5.1. Use of Antagonistic Microorganisms against PPF

Global trends are shifting away from the usage of agrochemicals to treat plant diseases. Presently, extensive research is underway to discover safe, environmentally acceptable, and efficient substitutes for synthetic chemical fungicides. The aim is to combat crop diseases in the field, which cause significant economic losses, and reduce decay loss in harvested commodities. Biological control, also known as biocontrol, emerges as the most suitable non-chemical approach to managing pests and pathogens in organic farming. It is highly specialized, environmentally safe, sustainable, and profitable [2]. Any reduction in the quantity or impact of pathogens (disease-producing activity) achieved by triggering biological mechanisms or the action of naturally occurring or introduced antagonists and modifications to the microenvironment favoring antagonist activity are considered biological control strategies for plant diseases [2,139].

Microbial BCAs (Biological Control Agents), typically derived from fungal or bacterial strains isolated from the phyllosphere, endosphere, or rhizosphere, play a vital role in controlling plant-harming microorganisms [2]. These agents preclude pathogens from infecting or establishing themselves in host plants. It is widely acknowledged that effective control methods primarily target pathogens. Antagonists employ various direct and indirect modes of action to manage biological diseases (Table 4) [2].

However, BCAs can encompass bacterial, fungal, or viral agents. Among bacterial BCAs, *Bacillus* and *Pseudomonas* are the most commonly used [139,140,141,142,143,144]. Within fungal BCAs, *Trichoderma* stands out as the most extensively studied genus, showing the greatest potential against PPF, with 25 BCAs identified [1].

#### 5.1.1. Use of Antagonistic Bacteria

The contribution of beneficial bacteria to crop disease resistance and plant growth has been the subject of numerous studies. Many bacteria from different genera have been identified as BCAs to manage a variety of diseases in major crops, including *Bacillus*, *Paenibacillus*, *Agrobacterium*, *Bradyrhizobium*, *Acinetobacter*, *Azospirillum*, *Azotobacter*, *Pseudomonas*, *Rhizobium*, and *Streptomyces* [2,18,139]. The application of beneficial bacteria can enhance plant development and manage a range of bacterial, fungal, and nematode diseases without endangering the environment. Through the formation of plant–microbial interactions in the rhizosphere, beneficial bacteria produce biofilms and secondary compounds such as fengycin, iturin, bacillomycin, and surfactin, which reduce the population of plant pathogens [2,139]. Lipopeptides like fengycin, iturin, pumilacidin, mixirin, and surfactin exhibit antifungal properties and act against pathogenic fungi in rhizospheres [16,139,144]. It has been observed that biocontrol bacteria, such as *Bacillus* spp. and *Pseudomonas* spp., are effective against a variety of phytopathogens in major crops. *B. velezensis* isolates have been found to contain numerous biosynthetic gene clusters involved in the production of secondary metabolites [139,144]. Furthermore, *B. subtilis* has been shown to play a significant role in the management of many agricultural diseases [142,145]. A potential BCA for Fusarium wilt of watermelon is *B. subtilis* IBFCBF-4 [142]. *Erysiphe cichoracearum*-caused powdery mildew severely reduces tobacco leaf yield and quality, often resulting in significant financial losses upon its appearance. Lipopeptides, particularly bacillomycin D and fengycin, produced by *B. amyloliquefaciens* YN201732, exhibit excellent biocontrol activity against the fungal disease *E. cichoracearum* [146]. When accompanied by other *Bacillus* isolates, seed treatment comprising *B. subtilis* BY-2 effectively suppresses *S. sclerotiorum* on oilseed rape [141]. Additionally, various rhizobacteria have shown the potential to control plant pathogens by inducing the production of different volatile organic compounds in plants [147]. *Stenotrophomonas maltophilia* UPMKH2, a rhizobacterium, effectively suppresses blast disease, enhances yield attributes, and induces systemic resistance against blast disease in rice [147]. Table 5 provides details of different bacterial BCAs found to be effective against various PPF.

#### 5.1.2. Use of Antagonistic Fungi

Fungal antagonists play a crucial role in managing plant diseases and infections [153]. Fungi are cited as effective biocontrol methods, tactics, and approaches employed in plant disease management, especially as modern agricultural practices aim to reduce the use of chemically manufactured pesticides [1,8]. Due to fungi’s short generation time, high rates of sexual and asexual reproduction, and target specificity, the potential for using fungal BCAs against plant infections has significantly increased. Moreover, they can persist in the environment without a host by switching from parasitism to saprotrophism, thus promoting sustainability [1,153]. The genus that has shown the most promise in treating a variety of plant fungal infections is *Trichoderma*. Other fungal taxa proven to be effective against various PPF include *Alternaria*, *Aspergillus*, *Candida*, *Fusarium*, *Penicillium*, *Pichia*, *Pythium*, *Talaromyces*, and *Verticillium* [1,153]. It has been discovered that *Penicillium linzhiense*, a novel fungal species, is efficient against *Pyricularia oryzae*, an economically significant or emerging fungal pathogen [154]. When the biocontrol agent and the fungicide are compatible, using a BCA in conjunction with a synthetic fungicide or physical additive, either concurrently or successively, should enhance disease suppression [153,155]. Table 6 lists the numerous fungal BCAs proven to be successful in combating various PPF.

#### 5.1.3. Use of Genetically Modified (GM) Organisms

With the advent of different molecular tools, like genomics, genetic engineering, and recombinant DNA techniques, the biological control of plant diseases using fungal BCAs has significantly advanced in recent years. These precise methods have been employed to enhance fungal strains for agro-industrial operations. The creation of novel plant pathogen-resistant crop varieties or clones presents a generally accepted and perhaps long-term control strategy. Furthermore, extensive research has been conducted to identify the genetic characteristics of fungal antagonists and determine their potential for improving biocontrol performance [153,164,165]. For example, the genome of *T. virens* was modified to incorporate multiple lytic enzyme-encoding genes. This resulted in a strain that secretes a mixture of chitinases, proteases, and β-1,3- and β-1,6-glucanases, leading to significantly increased inhibition of the pathogens *Pythium ultimum*, *R. solani*, and *Rhizopus oryzae* [166]. Through insertional mutagenesis via restriction enzyme-mediated integration transformation, a reduced-pathogenic mutant of the avocado fruit pathogen *Colletotrichum gloeosporioides* was developed. This mutant can be used to biologically control anthracnose, which is caused by *C. gloeosporioides* [167]. Natural interactions between plants and pathogens, or between pathogens and BCAs at the molecular level, help to elucidate the disease progression and the antagonistic mechanisms of BCAs [168]. This understanding further facilitates the development and utilization of genetically modified (GM) BCAs for specific and effective applications (Table 7).

Many R genes have been cloned and inserted into different types of plants. Transgenic potatoes expressing the wild potato R gene RB or Rpi-vnt1.1 demonstrate strong field resistance to *P. infestans*, the causative agent of potato late blight [179,180,181]. Molecular stacking is another genetic manipulation technique wherein researchers assemble multiple R gene cassettes on a single plasmid and then deliver this R gene cluster en bloc at a single genetic locus through plant transformation [182]. In an experiment using agrobacterium-based transformation of a susceptible cultivar, three broad-spectrum potato late blight R genes—Rpi-sto1, Rpi-vnt1.1, and Rpi-blb3—were molecularly stacked. Under greenhouse conditions, the resulting triple gene transformants demonstrated broad-spectrum resistance to the causal agent of potato late blight, *P. infestans*, equivalent to the cumulative strain-specific resistance imparted by each of the three unique Rpi genes [183]. It is thought that the correct placing and timing of late blight R gene stacks in potatoes could reduce the crop’s fungicide usage by more than 80% [184].

The guided development of a precisely defined window of genomic DNA in vivo has recently become possible through the development of the CRISPR-based mutagenesis platform called EvolvR [185]. EvolvR utilizes the mutagenesis activity of a fusion of the Cas9 nickase and an error-prone DNA polymerase to introduce nucleotide diversification at particular genomic areas defined by guide RNAs [185]. Recently, the CRISPR/Cas9 system has been applied to improve disease resistance in various crops, such as tomato, rice, cocoa, wheat, and grape. Modifying the genomes of fungi and oomycete pathogens can offer novel approaches to managing plant diseases, in addition to crop genome editing [186].

Using the CRISPR/Cas9 system, several methods are available for studying plant disease resistance. These methods include deleting, modifying, or introducing cis-elements in promoters; introducing specific mutations in coding regions via homology-directed repair (HDR); changing the amino acids in plant surface receptor proteins to evade secreted pathogen effectors (e.g., AtBAK1); deleting negative regulators of plant defense responses (e.g., TcNPR3); or modifying central regulators of defense response (e.g., BnWRKY70) [187,188,189,190,191]. The most extensively researched gene for resistance to fungal infections is the susceptibility gene, also known as the mildew resistance locus O (MLO) [186,191]. For instance, the *SlMlo1* gene in tomato plants has been knocked out using CRISPR/Cas9. The resulting knock-out mutants provided resistance to the powdery mildew fungus *Oidium neolycopersici* without further undesirable phenotypic effects [192]. Additionally, one of the three MLO homoalleles, TaMLO-A1, demonstrated enhanced resistance to *B. graminis f.* sp. *tritici* infection in wheat plants modified using CRISPR/Cas9 [193]. In grapes, biallelic mutation mutant lines were produced by CRISPR/Cas9-mediated targeted mutagenesis of the transcription factor VvWRKY52, and the knock-out of VvWRKY52 increased resistance to gray mold disease caused by *B. cinerea* [194].

Utilizing CRISPR/Cas9 to edit the genomes of oomycete and fungal organisms can yield novel approaches to managing plant diseases. An example is the filamentous PPF *Claviceps purpurea*. The CRISPR/Cas9 technique was used for the targeted mutagenesis of genes (*pyr4* and *TrpE*) involved in tryptophan biosynthesis and pyrimidine biosynthesis in *C. purpurea*. This led to a decrease in auxin, a plant hormone normally generated by *C. purpurea*, preventing TrpE mutants from infecting rye plants and hindering the fungus’s capacity to colonize rye plants [195]. Another instance involves the use of the CRISPR/Cas9 system to knock out the pectin acetylesterases (*PlPAE4* and *PlPAE5*) from *Peronophythora litchi*, the causative agent of downy blossom blight. Knock-out mutants of *PLPAE5* were less able to invade hosts compared to the wild-type strain, indicating *PlPAE5*’s involvement in oomycete pathogenicity [196]. Homozygous PpalEPIC8 mutants were also created in the papaya pathogen *P. palmivorausing* using CRISPR/Cas9 technology. These mutants showed decreased pathogenicity on papaya fruits because the cysteine protease inhibitor PpalEPC8 was knocked out, leading to less inhibition of papain, a cysteine protease that provides protection against plant pathogens [196].

#### 5.1.4. Use of Viral Vectors/Phages

*S. sclerotiorum* hypovirulence-associated DNA virus 1 (SsHADV-1) infects *S. sclerotiorum* DT-8, its host, resulting in hypovirulence, a decreased growth rate, and other morphological alterations to the colony. Still unknown, though, are the mechanisms underlying this [197]. One of the best examples of multitrophic interactions is mycovirus-mediated hypovirulence, which has gained interest because of its potential for biocontrol. By preventing the fungal manufacture of the phytotoxin Altersolanol A, a mycovirus known as *Stemphylium lycopersici* alternavirus 1 (SlAV1) from the plant disease *Stemphylium lycopersici* causes hypovirulence and pigmentation loss. Enhancing plant resistance to virulent strains, the pathogen becomes a biocontrol agent by genomic integration and the expression of a critical *SlAV1* gene in the fungal host. This creates a viable and low-risk avenue for biocontrol to contain plant diseases [198]. Similarly, a replication initiation protein (Rep) and a coat protein (CP) are encoded by the *S. sclerotiorum* hypovirulence-associated DNA virus 1 (SsHADV-1), which was identified from the PPF *S. sclerotiorum* [199]. The potential to investigate fungal viruses as useful tools for the molecular manipulation of fungi and the control of plant diseases is increased by many PPF, such as those that can transport mycovirus from one plant to another [200,201]. To counteract PPF, phages have been employed as BCAs. Peptidoglycan hydrolases and lysins from the phages Atu_ph02 and Atu_ph03 can lyse *Agrobacterium tumefaciens* (which causes crown gall disease) by preventing cell division [202].

### 5.2. Use of AMPs

**Figure 2:**
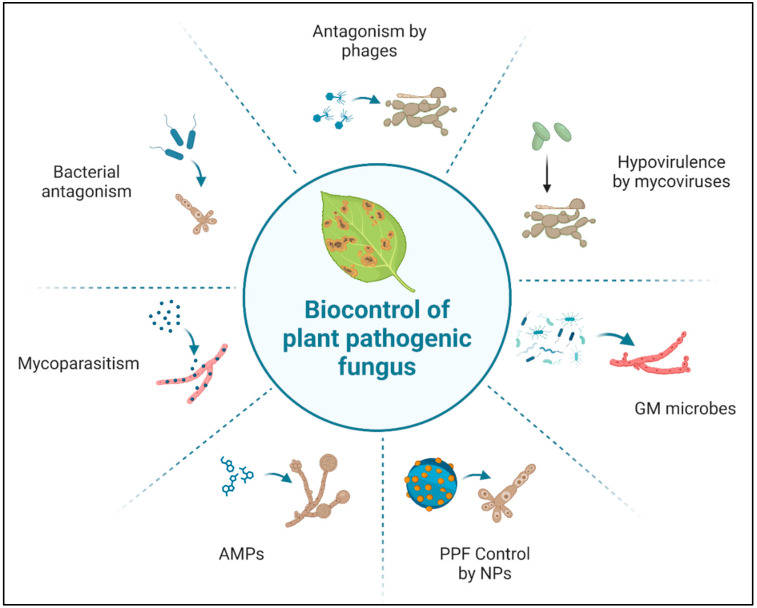
Mode of action of different biopesticides. AMPs, antimicrobial peptides; NPs, nanoparticles; PPF, plant pathogenic fungus; GM, genetically modified. This figure was created using BioRender software.

As a part of their defense mechanism, bacteria, fungi, and plants all produce various AMPs that are effective against various kinds of PPF (Figure 2). Beneficial bacteria generate a wide variety of natural chemicals to effectively treat different plant diseases. It has been discovered that certain *Bacillus* species produce mycosubtilin, subtilisin, sublancin, bacilysin, chlorotetain, mycobacillin, rhizocticins, bacillaene, difficidin, iturin A, bacillomycin, fengycin, bacilysin, and mersacidin, which are helpful in managing different PPF [139,142,144,203]. Numerous natural products, including extracellular enzymes like cellulase, chitinase, proteases, and beta-glucanase, as well as other antimicrobial substances like cyanide, phenazines, siderophores, and 2,4-diacetyl phloroglucinol, which inhibit numerous PPF, have also been reported to be produced by *Pseudomonas* species [151,152,153]. The majority of fungal plant diseases are managed by members of the fengycin and iturin families. These lipopeptides have been reported to cause harm to the hyphae and conidia of a variety of fungal pathogens, such as *F. graminearum* and *M. fructicola* [151,152,204]. Furthermore, several volatile organic compounds (VOCs) produced by bacteria, such as alcohol, aldehydes, ketones, hydrocarbons, acids, and terpenes, are responsible for regulating numerous PPF [16,18]. Numerous investigations have been conducted on the antibacterial qualities of volatile organic compounds (VOCs) against *S. sclerotiorum*, *M. fructicola*, *M. fructigena*, *B. cinerea*, and so on [16,18,152].

A variety of natural chemicals are produced by fungi, such as polyketides, aromatic compounds, non-ribosomal peptides, antibiotics, and heterocyclic metabolites. *Trichoderma* species generate several volatile organic compounds (VOCs) and AMPs, which are utilized as BCAs to manage various agricultural diseases [157,160,163,165]. *Trichoderma* spp. and Gliocladium virens are known to produce numerous antifungal chemicals, including gliovirin, viridiol, valinotrocin, viridin, gliotoxin, and heptelidic acid [205]. Studies have reported that *T. harzianum* strains T22 and TC39 produce antibiotics such as azaphilone, harzianopyridone, harzianolide, and 1-hydroxy-3-methylanthraquinone. These natural compounds inhibit the growth of plant pathogens such as *B. cinerea*, *R. solani*, *Leptosphaeria maculans*, *P. ultimum*, and *P. cinnamomin* [206]. *Chaetomium globosum*, in its culture filtrate, produces chaetoglobosin, an antibiotic used to control late blight disease in potatoes and reduce post-harvest illnesses in various fruits [207].

Various defense mechanisms, such as soluble proteins and volatile organic compounds (VOCs) released by plants possessing antifungal and antibacterial properties, have evolved in their innate immune system to protect against phytopathogens [208,209,210,211]. These proteins have broad functions beyond their antifungal activity; they can limit fungal growth and infection through various methods [208]. Pathogenesis-related (PR) proteins, AMPs, and polygalacturonase-inhibiting (PI) proteins are among the antifungal proteins [208]. Plant proteins known as PR proteins play a dual role in biotic and abiotic stress mitigation. They frequently induce systemic acquired resistance in plants. Chitinase is one of the most well-known and researched plant antifungal proteins [212]. By lysing hyphal tips in fungi and dissolving chitin into its oligomers, it prevents fungal growth [212]. *R. solani* and *B. cinerea* are just two examples of the many phytopathogenic fungi against which chitinases exhibit potent antifungal activity [22,213]. Additionally, chitinase derived from microorganisms exhibits strong antifungal properties. Chitinase from *Trichosanthes dioica* was able to efficiently control *Trichoderma* spp. and *A. niger*. The majority of vegetative and reproductive plant tissues express plant defensins, another extensively researched PR protein, constitutively in the extracellular space [214]. These plant defense compounds are effective against several PPFs. For example, *Phaseolus vulgaris* beans’ defensin-like protein NRBAP maintained its antifungal action against *Mycosphaerella arachidicola* at temperatures as high as 100 °C and in the pH range of 1–13 [215]. Another class of PR proteins active against various PPFs is called plant protease inhibitors (PIs). In vitro, *Rhizoctania solani* and *Candida ablicans* are inhibited by potato peptide-G [216]. Similarly, the growth of fungi such as *B. cinerea*, *P. infestans*, *R. solani*, *F. solani*, and *F. oxysporum* can be inhibited by potato protease inhibitors I and II (PPI–I and PPI–II) [217,218]. A different study found that broad beans contain a Bowman–Birk-type trypsin–chymotrypsin inhibitor that can stop the growth of *B. cinerea*, *F. oxysporum*, and *M. arachidicola* with as little as 60 μg per plate [219]. Another protein of interest are AMPs. AMPs are involved in the innate immune system of plants. Different antifungal peptides (AFPs) are produced by plants [208]. Among them is tomato systemin. Research indicates that systemin travels through the phloem of plants, assisting in signaling amplification and enabling distal leaves to respond to wounds [220]. Furthermore, the late blight-causing pathogen *P. infestans* induces at least 50% fewer lesions in transgenic plants expressing prosystemin [221]. *B. cinerea* infection was successfully managed by spraying 100 pM of systemin onto grapevine and eggplant plants [222]. It has been demonstrated that the potato antimicrobial peptide Snakin-2 is effective in vitro against *B. cinerea* and several *Fusarium* species [223].

### 5.3. Use of Nano-Based Technologies

In addition to being essential micronutrients for plants, NPs can be utilized to directly reduce pathogen infections, thereby enhancing crop growth and yield, and potentially leading to further growth enhancement through nutritional benefits (Figure 2) [224,225]. Through their antifungal activities, NPs such as Ag, ZnO, Mg, Si, and TiO_2_ can directly inhibit crop diseases [224,225,226,227]. Late blight in potatoes caused by *P. infestans* was greatly suppressed by Ag_2_O and Ag_2_O/TiO_2_ nanostructures [224]. Potato plants treated with Ag_2_O and Ag_2_O/TiO_2_ NPs showed improved expression of defense genes and decreased disease severity in both greenhouse and field conditions [224]. One extensively researched NP known for its antifungal efficacy against various PPF is silver NP, sourced from various origins [225,227]. The synthesis of AgNPs (30 μg) induced by peanut shell extract demonstrated a 5 to 6 mm zone of inhibition against *P. infestans* and *P. capsici*, two agricultural fungal diseases [227]. AgNPs derived from rice leaf extract exhibited fungicidal action against *R. solani*, the rice pathogen responsible for sheath blight [228]. Treatment with AgNPs of 16.5 nm (20 μg/mL) improved the seedling vigor index in rice cultivars by 10 μg/mL and completely prevented the spread of illness caused by *R. solani* isolates [228]. According to a separate study, Ag-NPs, in conjunction with thiophanate methyl and fluazinam, were effective against isolates of *M. fructicola*, whether susceptible or resistant to benzimidazole. The mode of action of Ag-NPs is likely related to ATP metabolism rather than silver ion release [229]. Furthermore, the accumulation of *Fusarium* mycotoxin was significantly reduced by AgNPs (30 nm) [230]. Thus, AgNPs can be used to manage and treat various fungal infections affecting crops. ZnO-NPs could effectively and dose-dependently inhibit the proliferation of mycelial cells in isolates of *Alternaria alternative* that were resistant (BOSC-R) and sensitive (BOSC-S) to boscalid (BOSC) [226]. The fungitoxic activity of ZnO-NPs is attributed to either ROS generation or Zn+2 release. The enhanced toxic action of ZnO-NPs is likely related to ion homeostasis [226]. 2-Substituted-sulfanyl-5-amino/methyl-1,3,4-thiadiazoles were synthesized and evaluated against two phytopathogenic fungi, specifically, *R. bataticola* and *R. solani*. Nano-thiadiazole derivatives exhibited 2–4 times greater fungicidal efficacy against the test PPF, according to the study [231]. Rice pathogen *P. grisea* growth was inhibited by chitosan guar NPs (CGNPs) [232]. These studies collectively demonstrate NPs as promising alternatives to chemical fungicides for sustainable agriculture systems.

## 6. Conclusions and Future Perspectives

It is imperative to closely monitor the antibiotic resistance (AR) status of PPF. However, there is a lack of information on this subject, necessitating routine surveillance. Regulatory bodies should implement routine AR surveillance programs to provide direction, control antibiotic use, and monitor antibiotic resistance in the field. Additionally, the excessive use of fungicides can lead to the development of antifungal resistance in non-target fungal groups. Horizontal gene transfer (HGT) can contribute to resistance development against antifungal chemicals, allowing non-target fungal pathogens to spread resistance to target groups with prolonged or repeated exposure to antifungal chemicals. Therefore, monitoring antifungal resistance development among non-pathogenic and facultative pathogenic fungal populations is crucial. BCAs are rapidly emerging as excellent alternatives to chemical fungicides. However, certain obstacles hinder their widespread use. Ensuring that the selected biocontrol agent is specific to the target pathogen and does not harm beneficial organisms is a primary concern. Additionally, consideration must be given to the nutritional requirements of BCAs for their survival, as inadequate provision may compromise their biocontrol activity [233]. The stability and growth rate of BCAs under field conditions are also critical factors, as pathogens may outgrow BCAs, reducing their effectiveness. Thus, extensive research is needed to ascertain the specificity and efficacy of BCAs before field trials. Although there are limitations to using living microbes such as BCAs, antimicrobial peptides (AMPs) and nanoparticles (NPs) offer several advantages. They are less prone to resistance, highly specific, fast-acting, and do not require external support such as food or specific growth conditions. Combining the application of AMPs with NPs may yield revolutionary results in PPF biocontrol.

## Figures and Tables

**Table 1:** Crops with some common fungal diseases and recent research on antibiotics found to be effective against these PPF.

Crop	Diseases	Causative Agent	Recommended Antibiotic	References
Maize	Banded leaf and sheath blight	*R. solani*	Azoxystrobin + Difenconazole, Flutolanil, Pencycuron	[50,51]
	Southern corn leaf blight	*Biopolaris maydis*	Azoxystrobin + Difenconazole	[52]
	Sorghum downy mildew	*Pernosclespora sorghi*	Treat seeds with Metalaxyl + Mancozeb, Foliar spray Azoxystrobin + Difenconazole	[52]
	Rajasthan downy mildew	*Pernosclespora heteropogoni*	Treat seed with Apron 35 SD, foliar spray of Metalaxyl + Mancozeb	[52]
	Fusarium stalk rot	*Fusarium verticillioides*	Low dose of nitrogen and high dose of potassium	[52]
	Common rust	*P. sorghi*	Dithane M-45	[52]
	Curvularia leaf spot	*Curvularia lunata*	Foliar spray with Carbendazim + Mancozeb or Zineb	[52]
	Turcicum leaf blight/Northern corn leaf blight	*Exserohilum turcicum*	Mancozeb 75 WP + Hexaconazole 5 EC + Azoxystrobin + Difenconazole	[52]
Potato	Fusarium wilt	*F. oxysporum*	Fludioxonil	[53]
	Late blight	*Phytophthora infestans*	Cyazofamid, Mandipropamid, Oxathiapiprolin + Benthiavalicarb	[54]
	Cercospora leaf spot	*Cercospora* spp.	Benzovindiflupyr + Difenoconazole, Pydiflumetofen + Difenoconazole	[55]
Tomato	Fusarium wilt	*F. oxysporum*	Prochloraz, Bromuconazole, Benomyl, Carbendazim	[56,57]
	Tomato leaf mold	*Cladosporium fulvum*	Trifloxystrobin	[58]
Eggplant	Fusarium wilt	*F. oxysporum*	Trifloxystrobin + Tebuconazole, Difenconazole Carbendazim	[59]
	Cercospora leaf spot	*Cercospora* spp.	Benzovindiflupyr + Difenoconazole, Pydiflumetofen + Difenoconazole	[55]
Blackberry	Fusarium wilt	*F. oxysporum*	Prochloraz, Difenoconazole, Triaendazole	[60]
Onion	Neck rot	*Botrytis allii*	Fluopyram, Tebuconazole, Boscalid, Pyraclostrobin	[61]
	Black mold	*Aspergillus niger*	Amphotericin B	[62]
Pumpkin	Black rot	*Didymella bryoniae*	Chlorothalonil, Mancozeb	[63]
	Sclerotinia rot	*Sclerotinia sclerotiorum*	Fludioxonil, Cyprodinil, Isopyrazam	[64,65]
Sugarcane	Rhizoctonia root and crown rot	*R. solani*	Azoxystrobin + Difenoconazole	[50]
	Sugarcane smut	*Sporisorium scitamineum*	Methoxy ethyl mercury Chloride, Zineb, Captafol, Triadimefon	[66]
Wheat	Septoria leaf blotch	*Z. tritici*	Epoxiconazole + Metconazole, Prothioconazole + Tebuconazole	[67]
	Yellow rust	*P. striiformis*		
	Brown rust	*P. triticina*		
	Eyespot	*Oculimacula yallundae*, *O. acuformis*	Prochloraz, Prothioconazole, Boscalid	[68]
Rice	Rice blast	*M. oryzae*	Camptothecin	[69]
Soybean	Rust	*P. pachyrhizi*	Azoxystrobin + cyproconazole	[70]
Corn	Corn smut	*U. maydis*	There is no chemical control for this disease	

**Table 2:** The main classes of fungicides and their mode of action.

Fungicide Class	Example	Mode of Action	References
Polyenes	Amphotericin B, Candicidin, Hamycin, Natamycin, Nystatin, Rimocidin	Binds with sterols in the fungal cell membrane, principally ergosterol, causing monovalent ion (K^+^, Na^+^, H^+^, and Cl^−^) and small organic molecule leaks	[71]
Allylamines	Butenafine, Naftifine, Terbinafine	Inhibits ergosterol biosynthesis by inhibiting squalene epoxidase	[45]
Morpholine	Aldomorph, Fenpropimorph, Dodemorph, Tridemorph	Inhibits ergosterol synthesis by blocking Erg2 and Erg24, catalyzing ∆14-reductase and ∆8-∆7-isomerase	[44,45]
Anilinopyrimidine	Cyprodinil, Mepanipyrim, Pyrimethanil	Inhibits methionine synthesis and secretion of hydrolytic enzymes	[72]
Azole	Abafungin, Bifonazole, Butoconazole, Clotrimazole, Econazole, Fenticonazole, Ketoconazole, Miconazole, Oxiconazole, Sulconazole, Albaconazole, Finaconazole, Epoxiconazole, Fluconazole, Cyproconazole, Prothioconazole, Tebuconazole, Metconazole, Propiconazole, Tebuconazole	Inhibits ergosterol biosynthesis by inhibiting 14α-demethylase	[44,45,46]
Echinocandins	Anidulafungin, Caspofungin, Micafungin	Inhibits 1,3-Beta-glucan synthase, thereby inhibiting the creation of glucan in the fungal cell wall	[48]
Others	Carboxin, Benodanil, Flutolanil, Fenfuran, Fluxapryroxad, Fluxypyram, Thifluzamide, Furametpyr	Inhibits succinate dehydrogenase and fungal respiration by binding to the ubiquinone-binding site in the complex II of mitochondria	[73]
	Benomyl, Carbendazim, Flubendazole	Inhibits microtubule assembly	[74]
	Azoxystrobin, Mandestrobin, Pyraclostrobin, Kresoxim-Methyl, Dimoxystrobin, Famoxadone, Fluoxastrobin, Fenamidone, Pyribencarb	Inhibits mitochondrial respiration by binding to the quinol oxidation site of complex III	[75]

**Table 4:** Different types of mechanisms of action of beneficial microbes as BCAs.

Type	Mechanism	Example
Direct Mechanisms	Apoptosis	Restrict the growth of biotrophs or hemibiotrophs in plants
	Parasitism	*Bacillus subtilis*, *Bacillus velezensis*, *Pasteuria penetrans*, *Trichoderma virens*
	Cell wall degradation	Produce chitinases, glucanases, proteases
	Antibiotic biosynthesis	2,4-diacetylphloroglucinol, phenazines, cyclic lipopeptides, and polyketides
	Competition	Exudates/leachates consumption, siderophore scavenging, physical niche occupation
	N_2_ fixation	*Rhizobium* (formerly *Agrobacterium*), *Azospirillum*, *Azoarcus*, *Herbaspirillum*, *Cyanobacteria*, *Rhodobacter*, *Klebsiella*
Indirect Mechanisms	Induce hormone modulation	Cytokinin, gibberellin, salicylic acid, auxin, and ethylene
	Growth promotion	Growth-promoting bacteria (PGPB), arbuscular mycorrhizal fungi (AMF), and rhizobia by N_2_ fixation, solubilizing phosphate and potassium
	Trigger immune system	Salicylic acid, jasmonic acid, and ethylene
Adaptive Mechanisms	Drought stress	Microbial production of phytohormone, exopolysaccharides, and mineral uptake, induce stress response genes
	Iron uptake via siderophore	Induce high-affinity Fe transport systems
	UV stress	*Methylobacterium* spp. mitigation of UV stress in mung bean
	Biofilm formation	Induce plant growth and protect plants from phytopathogens
	Carbohydrate utilization	Plant immunity and sugar recovery are activated by microbe-mediated sugar leakage
	Modulation in gene expression	Induce and regulate genes like *CaPR-10*, *sHSP*, *P5CR*, and *Cadhn*
	Induction of host resistance	Contact with fungal cell walls, detection of pathogen-associated molecular patterns, phytohormone-mediated induction

**Table 5:** Various bacterial BCAs found to be effective against PPF.

Plant	Pathogens	Bacterial BCAs	Mode of Action	References
Maize	*S. sclerotiorum*	*B. velezensis* VM11	Produces volatile organic compounds (VOCs) reducing sclerotial production and mycelial growth	[18]
Tomato	*S. sclerotiorum*	*B. amyloliquefaciens* FZB42	Produces VOCs, especially fengycin	[16]
Tobacco	*E. cichoracearum*	*B. amyloliquefaciens* YN201732	Produces lipopeptides, especially bacillomycin D and fengycin	[146]
Banana	*Fusarium* spp.	*B. subtilis*	Biosynthesis of bioactive compounds like iturin A, bacillomycin, fengycin, surfactin, bacilysin, tasA, and mersacidin	[145]
Watermelon	*F. oxysporum*	*B. subtilis* IBFCBF-4	Biosynthesis of bioactive compounds like iturin A, bacillomycin, fengycin, surfactin, bacilysin, tasA, and mersacidin	[142]
Tabaco	*S. sclerotiorum*	*B. amyloliquefaciens* EZ1509	Produces lipopeptides, especially bacillomycin D and fengycin	[143]
Arabidopsis	*A. alternata*	*B. amyloliquefaciens*, *B. subtilis*	Produces lipopeptides, especially bacillomycin D and fengycin	[148]
Potato, Tomato	*F. oxysporum*			
Maize	*F. verticillioides*			
Mustard	*S. sclerotiorum*	*B. thuringiensis*	Produce various VOCs	[149]
Pumpkin	*S. rolfsii*	*Serratia marcescens*	Possesses chitinolytic activity by releasing chitinase	[150]
Maize	*Pantoea ananatis DZ-12*	*Pseudomonas protegens* Pf-5	Polyketide pyoluteorin-produced reactive oxygen species and lipopeptide orfamide A	[151]
Stone fruit	*M. fructicola*, *M. fructigena*	*Pseudomonas synxantha*	Product-diffusible toxic metabolites and VOCs	[152]
Rice	*M. oryzae*	*P. putida* BP25	2-methylpyrazine and 2-ethyl-3,6-dimethylpyrazine produced by BP-25 inhibits the growth of *M. oryzae*	[140]

**Table 6:** Various fungal BCAs found to be effective against PPF.

Plant	Pathogens	Disease	Fungal BCAs	Mode of Action	References
Onion	*Sclerotium cepivorum*	White rot	*T. asperellum*	Activate salicylic acid-dependent defense pathways (1 and 7 days), and ethylene-dependent defense pathways (at 21 days)	[156]
Bean	*S. sclerotiorum*	White mold	*T. harzianum* ESALQ-1306, *T. asperellum* BRM-29104		[157]
Lettuce	*Sclerotium* sp.	Steam rot	*Trichoderma* sp. T76-12/2	Produce β-1,3-glucanase	[158]
Maize	*F. graminearum*, *F. verticillioides*	Stalk rot, ear rot	*T. asperellum*	Accumulate of deoxynivalenol and fumonisin B1 decreased in the stem and ear of maize	[159]
Apple	*A. alternata*	Fusarium wilt	*T. rossicum*, *T. harzianum*	Increase jasmonic acid content and accelerate soil metabolic processes of plants	[160]
	*Fusarium* spp.				
	*Cytospora mandshurica*				
Garlic	*S. sclerotiorum*	White rot	*Candida labiduridarum* (Yeast)	Produce VOCs	[161]
Groundnut	*A. niger*	Collar rot	*T. viride*	Elevate activities of the antioxidant enzymes, viz., superoxide dismutases, guaiacol peroxidase, and ascorbate peroxidase, in response to pathogen infection in treated plants	[162]
Mango	*Colletrotrichum gloeosporiodes*	Anthracnose	*T. asperellum* T8a	Produce lytic enzymes cellulases, chitinase, and glucanase	[163]

**Table 7:** Some fungal genes and their contributions investigated in plant–fungus interaction studies. This table was prepared by a modification from Raman et al. 2020 [168].

Plant	Pathogens	Gene Product	Disease	References
Arabidopsis	*Erysiphe orontii*	Receptor-like kinase	Powdery mildew	[169]
	*Alternaria brassicicola*, *B. cinerea*	Expansin	Gray mold/rot, leaf spot	[170]
	*Golovinomyces orontii*	Membrane-attached protein	Powdery mildew	[171]
	*Hyaloperonospora arabidopsidis*	ADP ribosylation factor—GTPase activating factor	Downy mildew	[172]
Maize	*Bipolaris maydis*/*Cochliobolus heterostrophus*	Mitochondrial transmembrane protein	Southern corn leaf blight	[172]
	*B. graminis*	Long-chain aldehyde synthesis	Powdery mildew	[173]
Tomato	*B. cinerea*	Polygalacturonase and expansin	Gray mold/rot	[173]
		ABA aldehyde oxidase		[174]
	*Leveillula taurica*	Membrane-anchored protein	Powdery mildew	[175]
	*F. oxysporum*	Lipid transfer protein	Fusarium wilt	[176]
Rice	*M. oryzae*	Transcription factor WRKY	Rice blast	[177,178]
